# Modelling of the Energy Depletion Process and Battery Depletion Attacks for Battery-Powered Internet of Things (IoT) Devices

**DOI:** 10.3390/s23136183

**Published:** 2023-07-06

**Authors:** Godlove Suila Kuaban, Erol Gelenbe, Tadeusz Czachórski, Piotr Czekalski, Julius Kewir Tangka

**Affiliations:** 1 Institute of Theoretical and Applied Informatics, Polish Academy of Sciences, Bałtycka 5, 44-100 Gliwice, Poland; seg@iitis.pl (E.G.); tadek@iitis.pl (T.C.); 2 Department of Computer Graphics, Vision and Digital System, Faculty of Automatic Control, Electronics and Computer Science, Silesian University of Technology, Akademicka 16, 44-100 Gliwice, Poland; piotr.czekalski@polsl.pl; 3 Renewable Energy Laboratory, University of Dschang, Dschang, Cameroon; tangkajkfr@yahoo.fr

**Keywords:** Internet of Things (IoT), energy depletion attacks (EDA), energy depletion process (EDP), diffusion approximation, battery modelling

## Abstract

The Internet of Things (IoT) is transforming almost every industry, including agriculture, food processing, health care, oil and gas, environmental protection, transportation and logistics, manufacturing, home automation, and safety. Cost-effective, small-sized batteries are often used to power IoT devices being deployed with limited energy capacity. The limited energy capacity of IoT devices makes them vulnerable to battery depletion attacks designed to exhaust the energy stored in the battery rapidly and eventually shut down the device. In designing and deploying IoT devices, the battery and device specifications should be chosen in such a way as to ensure a long lifetime of the device. This paper proposes diffusion approximation as a mathematical framework for modelling the energy depletion process in IoT batteries. We applied diffusion or Brownian motion processes to model the energy depletion of a battery of an IoT device. We used this model to obtain the probability density function, mean, variance, and probability of the lifetime of an IoT device. Furthermore, we studied the influence of active power consumption, sleep time, and battery capacity on the probability density function, mean, and probability of the lifetime of an IoT device. We modelled ghost energy depletion attacks and their impact on the lifetime of IoT devices. We used numerical examples to study the influence of battery depletion attacks on the distribution of the lifetime of an IoT device. We also introduced an energy threshold after which the device’s battery should be replaced in order to ensure that the battery is not completely drained before it is replaced.

## 1. Introduction

Hundreds of billions of wireless sensor devices are expected to be connected to the Internet through IoT access networks. Small, cost-effective batteries with limited energy capacity will power most of these sensors [1]. Recent advances in low-cost and low-power IoT technologies have enabled the cost-effective, energy-efficient, data-driven, and flexible automation of cyber-physical systems. However, the energy required to power these systems and related infrastructures will be enormous when hundreds of billions of IoT devices are connected to the Internet. In order to ensure that IoT devices are small and cheap for commercial deployment in large numbers, they are generally designed to have limited battery capacity, low computational power, limited memory, and use low-power communication protocols [2,3]. Therefore, when choosing batteries for IoT devices, it is essential to consider constraints such as cost, size, and capacity (energy rating of the battery in Wh, which determines the lifetime of the IoT device).

Furthermore, the limited computational, communication, and energy resources in IoT devices make it challenging to deploy complex security mechanisms and implement traditional cryptographic algorithms, as they require non-constant execution time [4]. Some IoT manufacturers and service providers design or deploy IoT devices without appropriate security mechanisms, making them vulnerable to cyberattacks. Cyberattacks that could be launched against an IoT device include identity theft, eavesdropping, man-in-the-middle attacks, and energy depletion attacks. One of the components of an IoT device that malicious attackers often target is the small, limited-capacity battery used to power the IoT device [5]. This kind of attack is often called an energy depletion attack and is analysed in this paper. Therefore, the critical constraint in designing and deploying IoT devices and networks is establishing a reasonable tradeoff between power consumption, throughput, security, coverage area, battery lifetime, and financial cost.

The design specifications of an IoT device and those of the battery used to power the IoT device should be chosen in such a way as to ensure a longer lifetime while ensuring acceptable QoS and security of the device. The lifetime of an IoT device is the time required to deplete the battery’s energy completely. Mathematical modelling frameworks have been proposed to establish the relationship between the device and battery parameters with the lifetime of an IoT device. The authors in References [6,7] modelled the energy depletion process of the battery of an IoT device (without energy harvesting) as a pure death Markovian process (since energy is drawn from the battery and the battery is not recharged). They used their model to study the impact of energy depletion attacks on the lifetime of an IoT device. The limitation of their model is in the assumption that the time required to consume a unit amount of energy is exponentially distributed. We address this limitation by proposing a model in which energy is treated as a continuous variable. The time required to consume a unit of energy from a battery must not necessarily be exponentially distributed (and could even be deterministic). The authors in Reference [8] discussed the use of diffusion approximation to model the dynamic changes in the energy content of the battery of an IoT device in the presence of an energy harvesting source that continuously replenishes the energy drawn from the battery.

In this paper, we applied a diffusion or Brownian motion process to model the energy consumption process of a battery of an IoT device. The diffusion process has been used in queueing models of computer systems and networks since 1970 when Gelenbe and Kobayashi introduced them, e.g., References [9,10,11]. Recently, they have been used in investigating modern network architectures, such as software-defined networks (SDN) [12,13]. We modelled ghost energy depletion attacks and their impact on the lifetime of IoT devices. Here, it helps us to determine the probability density function of the lifetime of an IoT device. We used numerical examples to study the influence of battery depletion attacks on the distribution of the lifetime of an IoT device. We also introduced an energy threshold in our model after which the battery of the device should be replaced to ensure that the battery is not completely drained before replacement. The rest of this paper is arranged as follows: Section 2 contains a brief overview of the power consumption of an IoT device and a review of stochastic modelling of the battery of an IoT device. Section 3 contains a review of energy depletion attacks in IoT networks. Section 4 contains the diffusion approximation modelling of the battery of an IoT device. Section 5 contains some numerical examples, and we conclude the paper in Section 6.

## 2. Energy Consumption Models for IoT Devices

**Figure 1:**
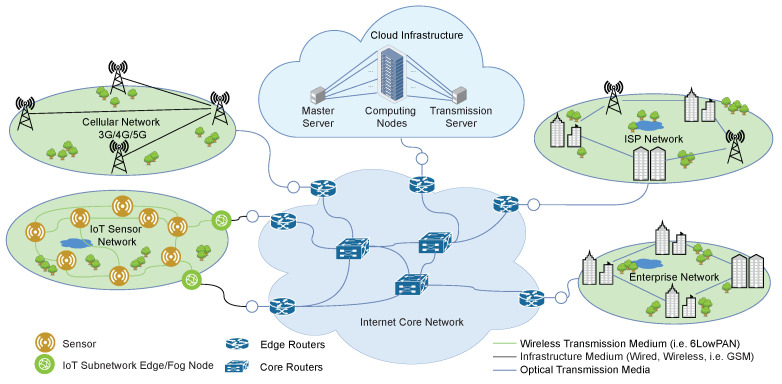
A simplified architectural model for the Internet of Things.

An IoT device consists of a sensing (data acquisition) unit, an actuator unit, a processing unit, a storage unit, a communication module, a power supply unit, and an energy storage system (battery). The sensors capture the desired physical data from the environment and convert them into digital information which may be partially processed by the IoT device or transmitted to fog computing servers for lightweight analysis or to a cloud computing data centre for advanced analysis. The analysis results could be returned to the IoT devices attached to actuators to control cyber–physical systems. Actuators receive digital signals and convert them into physical actions to drive or manipulate cyber–physical systems. A simplified architectural model of an IoT deployment is shown in Figure 1.

### 2.1. Power Consumption of an IoT Device

**Figure 2:**
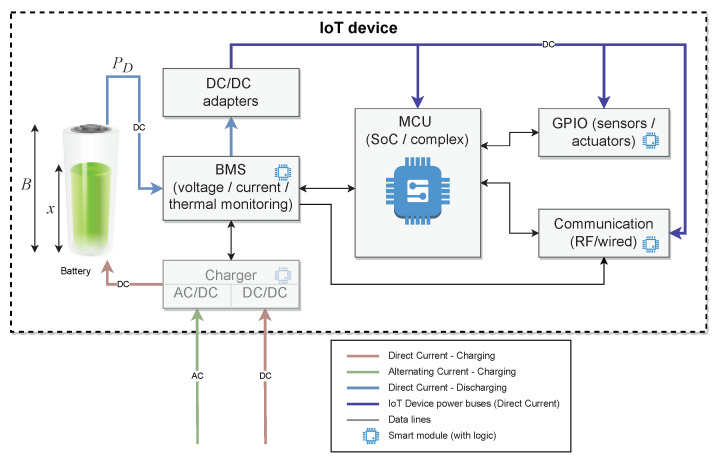
The architecture of an IoT device.

A simplified abstract architecture of an IoT device is shown in Figure 2. The core part of powering the IoT device is a BMS (battery management system) responsible for monitoring the battery state. The BMS constantly monitors voltage and current, thus calculating power drained, and monitors battery ageing via measuring remaining capacity on a charge/discharge cycle and the internal resistance and thermal profile of the battery cells. The BMS is responsible for checking critical battery conditions and informing MCU, eventually independently shutting down the power to prevent damage. This communication is bidirectional as MCU, and thus a device’s logic can monitor the current battery condition.

In some IoT devices, the BMS informs the communication processor independently (RF, wireless) on current battery conditions and power saving modes. In most constructions, this is achieved indirectly with the device logic via MCU.

As most IoT devices use rechargeable lithium-based batteries (lithium-ion, lithium-polymer, and their variations, i.e., Li-Fe), eventually, nickel–metal hydride batteries all require intelligent chargers that can be powered with AC or DC sources (i.e., solar panels or piezoelectric energy harvesters). As the voltage of such batteries varies substantially (i.e., a LiPo fully charged single cell is 4.2 V while discharged is 3.3 V), there is a need to use DC/DC adapters to stabilise voltage and provide a suitable one. Using a separate DC source (separate DC/DC adapter) for actuators’ like servos or motors is common. This is because of the possible inference of inductive actuators and their impact on the MCU and sensors’ work stability. Furthermore, many actuators require a different voltage, usually higher than MCU and sensors.

Details about the energy consumption profile of IoT devices are essential to model the energy depletion process of IoT batteries. The energy consumption profile of IoT devices depends on the energy-saving mechanisms deployed to minimise the energy consumption of IoT devices (e.g., use of low-power communication protocols, use of low-power microcontroller processing units, and configuring sleep modes to minimise the energy consumption of the device). These mechanisms significantly influence the device’s lifetime (the time required to completely deplete the energy stored in the IoT battery and shut down the device), a vital performance metric for the IoT device.

During the lifetime of the device, the device can either be in active mode (gathering data from the environment using sensors, manipulating physical systems using actuators, performing lightweight processing, or communicating with other devices or with an access point) or in sleep mode (in a sleep mode the energy consumption of the device is minimum or negligible). The power consumption models considered in this paper are inspired by the model developed in References [14,15,16]. They computed the average power consumption of an IoT device during its lifetime by summing the average power consumption of the device in the active mode and the average power consumption in the sleep mode. They derived the average power consumption in the active mode by calculating the power consumed by each module or component of the device (sensor/actuation modules, microcontroller units, communication units, and other electronic components). Following a similar approach, we derived the average power consumption of an IoT device, taking into consideration the fraction of time that the device spent in the active mode (the duty cycle ratio) [16],

$$R_{ACT}=\frac{T_{ACT}}{T_{ACT}+T_{SLEEP}}$$

where $T_{ACT}$ is the total time spent by the IoT device in the active mode, and $T_{SLEEP}$ is the total time spent in the sleep mode by the device.

The total power consumption of an IoT device is the sum of the power consumption of various IoT components, including the sensing units, the actuator unit, the microcontroller units, the communication unit, and the security unit. The average total power consumed by an IoT device is

(1) $$P_{D}=R_{ACT}\cdot \left(P_{SAU}+P_{MCU}+P_{Comm}+P_{Elec}\right)+R_{SLEEP}\cdot P_{SLEEP},$$

where $R_{ACT}$ is the fraction of time that an IoT device spends in the active mode, $R_{SLEEP}$ is the fraction of time the device spends in the sleep mode ($R_{ACT}+R_{SLEEP}=1$), $P_{SAU}$ is the average power consumed by the sensing and actuation units; $P_{MCU}$ is the average power consumed by the microcontroller unit; $P_{Comm}$ is the average power consumed by the communication module; $P_{Elec}$ is the average power consumed by other electronic components when the device is in the active mode; and $P_{SLEEP}$ is the average power consumption of the IoT device in the sleep mode.

The authors in Reference [17] modelled the energy consumption of an IoT node. They considered the energy consumed during an IoT device’s sensing, processing, and communication processes. A significant proportion of the energy is used to transmit and receive IoT packets. It depends on the IoT packet size, channel capacity, and the environmental factors that influence signal propagation through wireless transmission media. The authors in [1] proposed an analytical framework for modelling the energy consumption of an IoT device for a cellular IoT network (e.g., NB-IoT) and determined the lower bound for the energy consumption of the IoT device.

The most important requirements when designing and planning IoT devices and networks include cost, lifetime [18], and reliability. The lifetime of an IoT device depends on the capacity of the battery used to power the IoT device and on the energy drawn from the battery to power the IoT device. The battery’s capacity increases with cost, and some IoT applications require the deployment of tens, hundreds, or even thousands of battery-powered IoT devices, making cost a significant constraint to consider when designing and deploying IoT devices and networks. The device’s lifetime is the time required to completely deplete the energy stored in the battery and shut down the device. It could be increased by increasing the battery’s capacity or reducing the power consumed by the IoT device. The energy consumption of the IoT devices is kept at a minimum level by using an energy-efficient microcontroller, using a low-power communication protocol that keeps the device in sleep mode (energy-saving state) most of the time, or using lightweight energy-efficient security mechanisms. The authors in Reference [19] conducted a comprehensive measurement study of the energy consumption of NB-IoT devices in order to determine the factors that influence energy consumption and battery energy depletion. The authors found that NB-IoT’s energy consumption largely depends on the communication model, signal quality, energy-saving enhancements, and packet size.

### 2.2. Modelling of the Expected Lifetime of an IoT Device

The lifetime of an IoT device can be estimated using empirical methods or mathematical modelling. Empirical approaches require time and resources to set up the test bed for experiments. The authors in Reference [20] presented the first attempt to empirically estimate the lifetime of a battery-powered NB-IoT device using power consumption measurements. The authors in Reference [14] proposed a modelling and experimental framework for estimating the lifetime of battery-powered NB-IoT and LTE-M devices using energy consumption profiles. The expected lifetime of an IoT device is [14]

(2) $$L=\frac{B\cdot F_{bat}}{P_{D}}.$$

$F_{bat}$ is the battery safety factor that accounts for self-discharging, and *B* is the energy rating of the battery (in Wh). The average power $P_{D}$ required to power all the components of an IoT device could be estimated empirically or using theoretical power profile models for IoT. Since the IoT device is assumed to be either in active mode (data acquisition, processing, security, and communication) or sleep mode (the energy-saving state with the radio transceiver turned off), we derive the expected lifetime of the IoT device by substituting Equation (1) in Equation (3). Thus, the expected lifetime of the IoT device is

(3) $$L=\frac{B\cdot F_{bat}}{R_{ACT}\cdot P_{ACT}+R_{SLEEP}\cdot P_{SLEEP}}$$

where $P_{ACT}=P_{SAU}+P_{MCU}+P_{Comm}+P_{SEC}$ is the average power consumed by the IoT in active mode. By reducing the time spent by the device in the active mode or by increasing the time spent by the device in the sleep mode, the device’s lifetime can be prolonged. If the fraction of time that an IoT device spends in the active mode is $R_{ACT}=T_{ACT}/T_{D}$, and the fraction of time spent in the sleep mode is $R_{SLEEP}=1-R_{ACT}=T_{SLEEP}/T_{D}$, then Equation (4), as a function of $T_{ACT}$, $T_{SLEEP}$, and $T_{D}$, becomes:

(4) $$L=\frac{T_{D}\cdot B\cdot F_{bat}}{T_{ACT}\cdot P_{ACT}+T_{SLEEP}\cdot P_{SLEEP}}$$

where $T_{D}=T_{ACT}+T_{SLEEP}$. If the battery safety factor, $SF_{bat}=1$, then the expected lifetime of the IoT device given in Equations (2)–(4) becomes

(5) $$L=\frac{T_{D}\cdot B}{T_{ACT}\cdot P_{ACT}+T_{SLEEP}\cdot P_{SLEEP}}$$

which is the well-known formula for the expected lifetime of an IoT device, e.g., see References [19,20].

We know that the average total power delivered to the IoT device from the battery, $P_{D}=P_{ACT}+P_{SLEEP}$ can be expressed in terms of the average total current drawn from the battery, $I_{D}$, and the battery output voltage, $v_{o}$, as $P_{D}=I\cdot v_{o}$. The energy rating, *B* (in Wh), of the battery can also be expressed in terms of the charge rating, *Q* (in Ah), of the battery and the battery output voltage, $v_{o}$, as $B=Q\cdot v_{o}$. Therefore, by dividing the numerator and denominator of Equations (2)–(5) by the output voltage $v_{o}$, the lifetime of the IoT device as a function of the charge rating of the battery *Q* and the total average current delivered to the IoT device from the battery $I_{D}$ is

(6) $$L=\left\{\begin{array}{ccc} \frac{T_{D}\cdot Q\cdot F_{bat}}{T_{ACT}\cdot I_{ACT}+T_{SLEEP}\cdot I_{SLEEP}} & \;\;\;\;\;\;\;\;SF_{bat}>1\;, & \\ \frac{T_{D}\cdot Q}{T_{ACT}\cdot I_{ACT}+T_{SLEEP}\cdot I_{SLEEP}} & \;\;\;\;\;\;\;\;SF_{bat}=1, \end{array}\right.$$

where $I_{D}=R_{ACT}\cdot I_{ACT}+R_{SLEEP}I_{SLEEP}$, $I_{ACT}$ is the average current delivered to the IoT device from the battery in the active mode, and $I_{SLEEP}$ is the average current delivered to the IoT device from the battery in the sleep mode. One of the misconceptions when choosing batteries for IoT devices is the assumption that batteries with a higher charge rating will guarantee a long lifetime for the IoT device. For alkaline batteries, which are the most cost-effective and commonly available batteries used to power electronic devices, the output voltage $v_{o}$ degrades very quickly, shortening the time required to drain the battery completely or the lifetime of the IoT device [21]. The authors in Reference [22] highlighted the difference between the battery capacity, *Q* in Ah, and the battery capacity, *B* in Wh. The charge rating of the battery, *Q*, is the total amount of electricity generated due to electrochemical reactions in the battery. At the same time, *B* is the total energy that the battery can deliver during the discharge process. The relationship between the charge rating of the battery, *Q*, and the discharge current may not be linear, as shown in Equation (6), but is exponential according to Peukert law [23]. An alternative relationship between the charge rating of the battery, *Q*, the average discharge current, $I_{D}$, and the battery discharge time (which is equivalent to the lifetime of the IoT device), *L*, can be deduced from the Peukert law [22,23] as

(7) $$L=Q\cdot I_{D}^{-k}=\frac{Q}{{\left[R_{ACT}\cdot I_{ACT}+R_{SLEEP}\cdot I_{SLEEP}\right]}^{k}}$$

where *k* is the Peukert constant, and its value lies between 1.1 and 1.3. Therefore, it is preferable to estimate the expected lifetime of the IoT device using the energy rating (in Wh) (e.g., (2)–(5)), rather than the charge rating as in Equation (6).

Therefore, in order to estimate the expected lifetime of an IoT device, it is necessary to estimate the average power consumption of the device (the rate at which energy is drawn from the battery) and the battery rating or the initial amount of energy in the battery (initially the battery should be charged to full capacity). It is important to use measurements to benchmark the energy consumption of IoT devices before they are used to estimate the lifetime of an IoT device. Any hardware and software implementation changes or energy depletion attacks that alter the supposed sleep time of the device may significantly reduce the expected lifetime of the IoT device determined during IoT network design and deployment.

On the other hand, mathematical modelling provides a faster technique for understanding the relationship between the battery parameters, the power consumption model of the IoT device, and the lifetime of the IoT device. Since it is possible that the energy drawn from the battery could vary stochastically, stochastic models such as Markovian models, fluid flow models, and diffusion approximation models have been used to estimate the model of the battery of sensor devices and to estimate their lifetime.

### 2.3. Stochastic Modelling of the Battery of an IoT Device

A practical approach in the analysis and optimisation of energy, and more broadly for the joint optimisation of energy and quality of service in computer systems and networks, named “energy packets”, was introduced by Gelenbe in 2011 and 2012 [24,25,26]. It conveniently represents energy in discrete units, where an energy packet is the minimum amount of energy required to transmit a single data packet or process a single job. This approach was initially applied to the optimisation of power flow in multiple-node computer networks [27] and joint work and energy in computer systems [28] and then to study the effects of energy leakage in battery-powered devices [29]. The model was applied to the study of sensor nodes [30] and also battery attacks in References [31,32].

Battery depletion models use mostly Markov chains, fluid flow approximation, and diffusion approximation. A good example of the first approach is presented in Reference [33], where a nonstationary continuous-time Markov process is used to describe the evolution in time of the energy states of a nanosensor’s mote. It is assumed that the energy-generating and -consuming processes are of the Poisson type. Each energy state corresponds to the energy needed to receive or transmit a certain number of packets. Transition rates between states are time-dependent and determined based on device performance analysis. Both transient and steady states are considered. The model, validated by simulation, is used to investigate the impact of the packet size and retransmission policy on the end-to-end successful packet delivery probability, end-to-end packet delay, and the throughput of wireless nanosensor networks.

In Markovian models, the times necessary to produce and consume a quantum of energy are exponentially distributed. In the fluid flow approach, the delivery and consumption of energy are described only by their time-dependent mean rates. The difference in the input and output rates determines the speed of changes in battery content. In References [34,35], the authors develop fluid flow models with rates driven by a Markov chain to analyse the battery lifetime of user equipment in LTE networks (first) and to evaluate various energy-management policies (second). Furthermore, in Reference [36] multi-regime fluid queue models where charging and discharging rates depend on the energy level are used to study the lifetime of a battery.

The advantage of using diffusion models to model the time evolution of the energy stored in the battery over traditional queuing theoretic models and fluid flow models is that it takes into account fluctuations in the amount of energy harvested from the environment and the fluctuations in the amount of energy drawn from the battery (energy consumed).

The fluid flow approximation may be seen as a simplified version of diffusion approximation. It is a first-order approximation based on mean values: time-dependent mean input rate and mean service time, giving the time-dependent mean queue length, or, in our case, the time-dependent energy content. Diffusion approximation is based on the first two moments of interarrival and service time distributions (second-order approximation), giving the distributions of energy content and time to depletion. Therefore, by definition, the fluid flow approach gives results inferior to diffusion approximation; however, its computations are simpler. They need the resolution of first-order ordinary differential equations, whereas the diffusion model uses second-order partial equations. We compared the errors introduced by both methods in, e.g., References [37,38].

The authors in References [39,40] propose a diffusion process to model the energy supply process from energy harvesting, storage, and consumption for a battery of a sensor node. Metrics such as the average time until the energy in the battery is depleted for a given battery capacity, workload, and energy harvesting characteristics, are derived. In particular, in Reference [39], a pure diffusion model represents the time evolution of the discharging and charging process of the battery for a wireless sensor node, providing performance metrics such as the average amount of energy present in the battery at a time, *t*, the failure rate of the wireless sensor node when the battery is completely discharged, and the steady-state solution of the model. It is sometimes essential to obtain the transient distribution of the amount of energy stored in the battery at time *t* and the distribution of the discharging time, which the authors in References [39,40] did not consider directly. However, several transient results, such as the expected time for the battery to empty, can be obtained from steady-state analysis of a slightly modified model that returns to its initial state each time the battery empties.

The authors in Reference [41] applied diffusion approximation to analyse the transient evolution of the charging and discharging process of a battery that is supplied by renewable energy and then used to supply network nodes in a wireless mesh network. Diffusion approximation was recently applied in Reference [42] for the optimisation of the energy of the battery of an unmanned aerial vehicle (e.g., drone) during its mission.

## 3. Energy Depletion Attacks in IoT Networks

Energy depletion attacks are designed to exhaust the energy stored in the battery of an IoT device. Reliable security mechanisms are complex and require appropriate computing resources, memory, and energy. The limited resources (e.g., memory, processing power, bandwidth, and battery) in IoT devices make it challenging to implement reliable security mechanisms in devices and networks. Furthermore, some IoT device manufacturers do not implement security futures to keep the cost low (to be competitive in the market) and to speed up their manufacturing process. However, much effort has been made to implement lightweight security mechanisms that optimise the limited IoT resources while providing the required security for IoT devices.

Energy depletion attacks are designed to increase the energy consumption of IoT devices, rapidly depleting the energy stored in the battery and eventually shutting down the IoT device. The authors in Reference [5] presented two types of battery depletion attacks: service requests and benign power attacks. In the service request power attack, the attacker continuously sends service requests to the target device, which keeps the IoT device awake for extended periods and rapidly depletes the battery energy. In a benign power attack, an attacker forces a compromised IoT device to execute energy-demanding tasks, rapidly depleting the battery energy continuously. The authors also proposed a network-based intrusion detection and prevention technique to detect and prevent battery depletion attacks. Therefore, energy depletion attacks are designed to increase the amount of time an IoT device spends in active mode or reduce the fraction of time that the device spends in sleep mode.

The secure communication process is the most energy-demanding in an IoT device. Low-power communication protocols that keep the IoT packet size as small as possible and keep the device in sleep mode for as long as possible are used to reduce the energy consumption of IoT devices. Some energy depletion attacks are designed to reduce the sleeping time of the IoT device. Adjusting some of the network parameters, such as the duty cycle, data rates, and packet size, can significantly deplete the battery of an IoT device and reduce its lifetime [43,44]. Furthermore, by inducing the device to transmit useless packets repeatedly, the device rapidly depletes the battery’s energy [45]. A malicious attacker could prevent an IoT device from entering sleep mode by manipulating its contention window size [46], sending massive amounts of packets to create more collisions to the ongoing transmissions [47], and overwhelming the IoT device by flooding it with packets without payload.

Energy depletion attacks could reduce the lifetime of IoT devices from years to days [2] and shut down an entire IoT network. The authors in [48] studied the impact of battery training attacks such as “hello” flooding, stretch attacks, and versioning on the energy consumption of IoT devices. The authors found that versioning is the most severe as it draws much energy from the battery, followed by packet flooding and “hello” attacks. The authors in Reference [3] analysed the impact of battery drain or energy depletion attacks on IoT devices. They designed and conducted DoS service attacks such as “hello” flooding and version number modification to demonstrate the impact of these attacks on the energy consumption of the IoT devices and rendered some of them unreachable. A similar demonstration was shown in Reference [45], where the authors configured some malicious nodes to intentionally generate and send large number of packets to legitimate nodes to excessively consume the energy resources of the nodes found along the forwarding path.

Unmanned arial vehicles (UAVs) are increasingly being adopted for commercial applications [49] such as agriculture, environmental management, supply chains, law enforcement, surveillance, and photography [50,51,52], and they were recently used for deliveries during the COVID-19 Pandemic [53] and to enforce the restrictions designed to slow the spread of the COVID-19 virus. A UAV could be considered an IoT system, especially when connected to the Internet (e.g., used as an access point in some IoT deployments requiring a temporary sensor network for a few hours). Like traditional wireless sensor devices, batteries power UAVs, making implementing sophisticated, reliable security mechanisms difficult. As a result, the security mechanisms implemented in UAVs are relatively weak and could be easily compromised. One such possible attack is the energy depletion attack designed to take control of the UAV and cause it to perform manoeuvres that consume more energy and rapidly drain the drone’s battery. Although most UAVs have battery monitoring and management systems that ensure the drone does not crash due to energy outages by initiating a return-to-home (RTH) mechanism. The RTH mechanism ensures that the drone returns home with a reasonable amount of energy to ensure a safe landing. However, battery depletion attacks could still cause the drone to crash while executing the RTH procedure, which could be catastrophic and result in a lawsuit. The authors in Reference [54] presented a framework for simulating and assessing battery depletion attacks on UAVs in crisis management systems.

It is essential to have techniques to detect energy depletion attacks and mitigate their impact. Most attack detection systems that detect energy depletion attacks in IoT networks are based on monitoring traffic characteristics and QoS metrics. The authors in Reference [3] proposed an intrusion detection system (IDS) that detects the presence of energy depletion attacks in IoT networks by monitoring packet characteristics and QoS metrics such as packet sending rate, interpacket interval, and the receive signal strength (RSS). They also demonstrated the use of firewalls to detect traffic coming from intrusions. It should be noted that not all energy depletion attacks degrade the quality of service. Some energy depletion attacks may increase service quality while gradually increasing the rate at which energy is drawn from the battery until the battery becomes empty and the device(s) are shut down [6]. The authors in Reference [55] proposed a lightweight anomaly detection model against energy depletion attacks on IoT networks. The model proposed by the authors is based on the analysis of statistical distance metrics to differentiate between normal and abnormal energy consumption in IoT devices.

Some attempts have been made to model the impact of energy depletion attacks on the performance and safety of battery-powered IoT devices. The authors in References [6,7] modelled the impact of energy depletion attacks on the performance of IoT devices. They investigated the impact of increasing energy consumption due to energy depletion attacks on the system survivability metric (the mean time to failure [MTTF]) for an IoT device under an energy depletion attack. The authors discussed the impact of energy depletion attacks which do not degrade the QoS or may improve the QoS while gradually draining the battery of the IoT device. The model proposed by the authors is based on the pure death Markovian process, which assumes that there is no continuous energy supply in the battery. However, the energy consumption process is exponentially distributed. The Markov assumption limits the proposed model. The authors in References [31,32] analysed the impact of battery depletion attacks on the lifetime of an IoT device. They provided mathematical models that can be used to estimate the effect of battery depletion attacks that attempt to deplete the limited amount of energy stored in the battery. The authors considered two cases, one with energy harvesting and the other where the node is supplied by a battery that must be regularly replaced when completely drained.

## 4. Modelling the Energy Depletion Process for a Battery of an IoT Device

In this section, we present stochastic models that are useful for analysing the energy depletion process of the battery of an IoT device. We present a Markovian model of the battery of an IoT device developed and used to analyse battery depletion attacks in References [6,7]. We propose a similar diffusion-based model of the battery of an IoT device. The diffusion model is also compared with the Markovian model.

### 4.1. Markovian Model of the Battery for IoT Devices

The Markovian model presented in this subsection is based on the pure death process discussed in References [6,7]. Suppose that the battery is initially charged to its full capacity *B*. Furthermore, suppose that the energy stored in the battery is quantised and that those fixed-sized energy units are drawn from the battery to power the IoT devices. It is assumed that the energy consumption process is exponentially distributed. Let N(t),t≥0 be a random process that represents the number of energy units present in the battery at time *t*, and the probability that there are *n* energy units in the battery be $P\left\{N\left(t\right)=n\right\}=P_{n}\left(t\right)$, where $P_{n}$ is the state probability of having *n* energy units in the battery. The time evolution of the discharging process of the battery can be described as a pure death Markovian process as

(8) $$\begin{array}{ccc} \frac{dP_{B}\left(t\right)}{dt} & = & -P_{B}\left(t\right)P_{D}\\ \frac{P_{n}\left(t\right)}{dt} & = & -P_{n-1}\left(t\right)P_{D}+P_{n+1}\left(t\right)P_{D}\;\;\;0<n<B\\ \frac{dP_{0}\left(t\right)}{dt} & = & P_{1}\left(t\right)P_{D}, \end{array}$$

where $P_{D}$ is the energy consumption per unit time [representing the transition rate between neighbouring states in the Markovian model in Equation (9)], $P_{B}$ is the probability that the battery is full, and $P_{0}$ represents the probability that the battery is empty. The solution of the set of equations in Equation (9) gives the state probability of the amount of energy present in the battery at time *t*, and it is given by Reference [56]

(9) $$P_{n}\left(t\right)=\frac{{\left(P_{D}t\right)}^{B-n}}{(B-n)!}e^{-P_{D}t},\;\;\;0<n\leq B.$$

Using the normalisation $\sum_{n=1}^{B}P_{n}\left(t\right)+P_{0}\left(t\right)=1$, the probability that the battery is empty at time *t* (service outage probability) during the backout period is

(10) $$P_{0}\left(t\right)=1-\sum_{n=1}^{B}\frac{{\left(P_{D}t\right)}^{B-n}}{(B-n)!}e^{-P_{D}t}.$$

**Figure 3:**
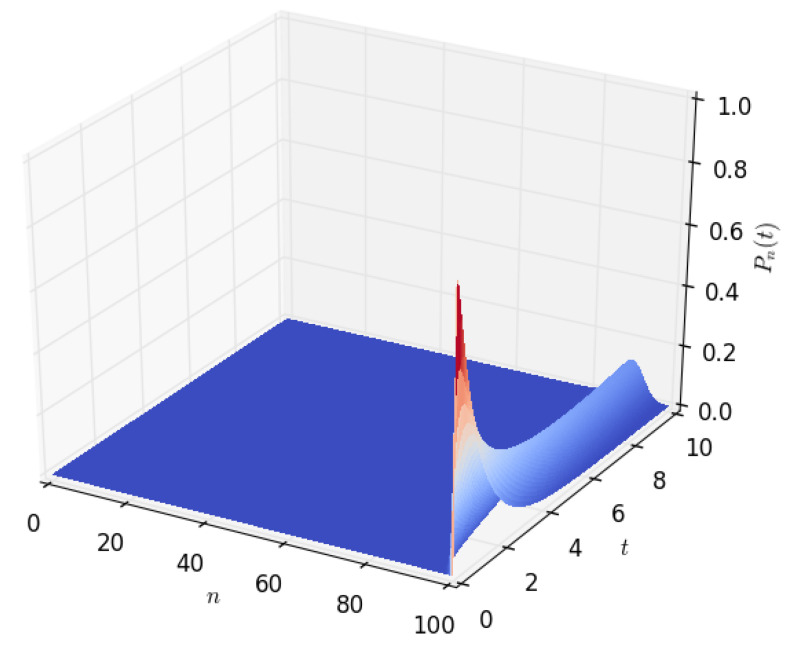
The distribution $P_{n}\left(t\right)$, of the amount of energy *n* in Wh present in the battery at time *t*, for t∈(0,10], n∈[0,100], B=100, and $P_{D}=1$.

**Figure 4:**
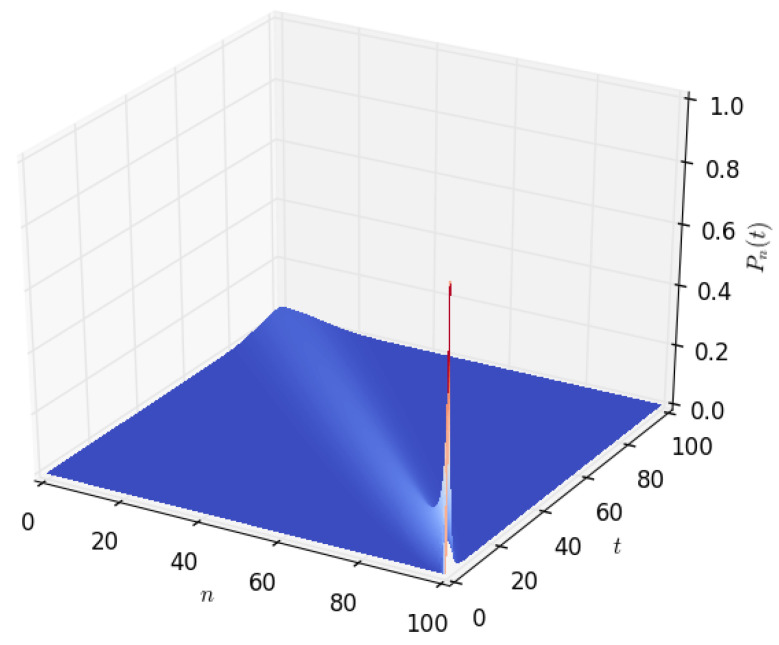
The distribution $P_{n}\left(t\right)$, of the amount of energy *n* in Wh present in the battery at time *t*, for t∈(0,100], n∈[0,100], B=100, and $P_{D}=1$.

Figure 3 and Figure 4 show the density of the number of energy units *n* present in the battery at time *t*. It starts with a sharp spike and then gradually decreases to zero. This is because, initially (at time t=0), we start with a battery fully charged to its capacity, *B*. The battery’s energy content gradually decreases with time and eventually reaches zero when all of the energy stored in the battery is completely depleted. As shown in Section 4.2 of this paper, a similar probability density can be obtained by modelling the battery using a diffusion process.

The time required to deplete the energy stored in the battery entirely is the first passage time of the Markov process from the state n=B at time t=0 to the state n=0 at time *t*; when the energy stored in the battery is completely depleted. It is the lifetime of the IoT device. Let the random variable *T* represent the time required to completely deplete the energy stored in the battery, where the first passage time process is T=inf{t>0:N(t)=0}. The distribution of the first passage time from a defined point n=B to n=0 is

(11) $$\gamma_{B,0}\left(t\right)={\left[P_{D}e^{-P_{D}t}\right]}^{*B}$$

where *B is *B*-fold convolution of a function with itself. The mean of the distribution (11) gives the mean of the time required completely depletes the amount of energy present in the battery and is given by

(12) $$E\left[T\right]=\frac{B}{P_{D}}=\frac{B}{R_{ACT}\cdot P_{ACT}+R_{SLEEP}\cdot P_{SLEEP}}.$$

Despite the assumption that the energy consumption process is exponentially distributed, the mean lifetime of the IoT device given in (12) is the same as the well-known expression for the lifetime of an IoT device given in Equation (2), for $F_{bat}=1$. The same expression can be derived using a diffusion approximation-based model. The diffusion approximation modelling approach removes the assumption that the energy consumption process at each stage (of the Markov process) should be exponentially distributed. Discretising the battery’s energy is also unnecessary, as diffusion is a continuous stochastic process.

### 4.2. Diffusion Approximation Model of the Battery for IoT Devices

Suppose that the cumulative amount of energy drawn from the battery to power the IoT device up to time *t* is $E_{D}\left(t\right)$; then, the amount of energy present in the battery at time *t* is

(13) $$E\left(t\right)=B-E_{D}\left(t\right)$$

The change in the amount of energy in the battery between time *t* and t+Δ is

(14) $$E\left(t+\Delta \right)-E\left(t\right)=\{E_{D}\left(t+\Delta \right)-E_{D}\left(t\right)\}$$

If we assume that as energy is drawn from the battery, the changes in the energy content of the battery ΔE(t)=E(t+Δ)−E(t) are normally distributed, then we can approximate the discharging process of the battery by a diffusion or Brownian motion process X(t) whose changes ΔX(t)=X(t+Δ)−X(t) are normally distributed with mean βΔt and variance αΔt where

$$\begin{array}{ccc} \beta & = & \lim_{\Delta t\to 0}\frac{E[X(t+\Delta t)-X(t)]}{\Delta t}\\ \alpha & = & \lim_{\Delta t\to 0}\frac{Var[X(t+\Delta t)-X(t)]}{\Delta t}. \end{array}$$

The changes in the energy content of the battery (during the discharging process) can then be described by the second-order partial differential diffusion equation

(15) $$\frac{\partial \psi (x,t;B)}{\partial t}=\frac{\alpha }{2}\frac{\partial^{2}\psi \left(x,t;B\right)}{\partial x^{2}}-\beta \frac{\partial \psi (x,t;B)}{\partial x}.$$

We represent the discharging process of the battery by a diffusion process that starts at X(0)=B (battery is fully charged at the beginning) and ends at X(t)=0 (when the battery is fully discharged) subject to the conditions

(16) $$\begin{array}{ccc} \psi (B,0;B) & = & \delta (B)\\ \psi (0,t;B) & = & 0\;,\;\;\;t\geq 0, \end{array}$$

where ψ(x,t,B) is the probability density function (pdf) that we have *x* amount of energy in the battery at time *t*, given that the discharging process started with x=B amount of energy in the battery—the initial condition ψ(x,0;B)=δ(B). The boundary condition ψ(0,t;B)=0 fort≥0 corresponds to an absorbing barrier at x=0, i.e.,

$$\lim_{x\to 0}f\left(x,t:B\right)=0;$$

the process is finished when it reaches the barrier. The probability that the process is at the barrier at time *t* corresponds to the probability that the battery is depleted at that time.

The solution of (15) with the above conditions is, e.g., [57],

(17) $$\psi \left(x,t;B\right)=\frac{e^{\frac{\beta }{\alpha }\left(x-B\right)-\frac{\beta^{2}}{2\alpha }t}}{\sqrt{2\pi \alpha t}}\left[e^{-\frac{{\left(x-B\right)}^{2}}{2\alpha t}}-e^{-\frac{{\left(x+B\right)}^{2}}{2\alpha t}}\right]\;.$$

If β<0, i.e., the value of the process is decreasing, then for t→∞, the probability that the process is at x=0 is equal to 1 and for all other *x*

$$\lim_{t\to \infty }\psi \left(x,t;B\right)=0.$$

The lifetime of the IoT device is the time required to deplete the energy stored in the battery completely, and it is the first passage time of the diffusion process from x=B (when the battery is fully charged) to x=0 (when the energy stored in the battery is completely depleted). Let the random variable *T* represent the lifetime of the IoT device, T=inf{t>0:X(t)=0}. The pdf $\gamma_{B,0}\left(t\right)$ of the first passage time of the diffusion process from x=B to x=0 is [57]

(18) $$\begin{array}{ccc} \gamma_{B,0}\left(t\right) & = & \int_{0+}^{\infty }\frac{\partial \psi (x,t;B)}{\partial t}dx=\\ & = & \int_{0+}^{\infty }\frac{\alpha }{2}\frac{\partial^{2}\psi \left(x,t;B\right)}{\partial x^{2}}-\beta \frac{\partial \psi (x,t;B)}{\partial x}dx=\\ & = & \lim_{x\to 0}[\frac{\alpha }{2}\frac{\partial \psi (x,t;B)}{\partial x}-\beta \psi \left(x,t;B)\right]=\frac{B}{\sqrt{2\pi \alpha t^{3}}}e^{-\frac{{\left(B+\beta t\right)}^{2}}{2\alpha t}}. \end{array}$$

The mean lifetime of the IoT device (or the mean time to failure) is

(19) $$\begin{array}{c} \mu_{T}=\int_{0}^{\infty }t\;\gamma_{B,0}\left(t\right)dt=\frac{-B}{\beta }. \end{array}$$

and the variance of the lifetime of the IoT device is

(20) $$\begin{array}{c} \sigma_{T}^{2}=\int_{0}^{\infty }t^{2}\;\gamma_{B,0}\left(t\right)dt-\mu_{T}^{2}=-\frac{B\alpha }{\beta^{3}}. \end{array}$$

If we consider a battery in which energy is stored at a mean rate of $P_{S}$ and energy is drawn from it at a mean rate $P_{D}$, then the energy in the battery changes at a mean rate $\beta =P_{S}-P_{D}$ [35]. The variance of these changes is $\alpha =C_{A}^{2}\cdot P_{S}+C_{B}^{2}\cdot P_{D}$, where $C_{A}^{2}$ and $C_{B}^{2}$ are the squared coefficients of variation of the process supplying energy to the battery and the process of drawing energy from the battery, respectively. However, in this paper, we assumed that energy is not supplied to the battery of the IoT device; that is, we assume that the device depends only on the energy stored in the battery during the deployment of the IoT device (i.e., $P_{S}=0$ and $C_{A}^{2}=0$). Therefore, the parameters of the diffusion process can be defined as $\beta =-(R_{ACT}\cdot P_{ACT}+R_{SLEEP}\cdot P_{SLEEP})$ and $\alpha =C_{B}^{2}\left(R_{ACT}\cdot P_{ACT}+R_{SLEEP}\cdot P_{SLEEP}\right)$, respectively. The diffusion parameter $\beta =-(R_{ACT}\cdot P_{ACT}+R_{SLEEP}\cdot P_{SLEEP})$ and $\alpha =C_{B}^{2}\left(R_{ACT}\cdot P_{ACT}+R_{SLEEP}\cdot P_{SLEEP}\right)$. Therefore, the expected lifetime of the IoT device or the mean time-to-failure (MTTF) becomes:

(21) $$\mu_{T}=\frac{B}{R_{ACT}\cdot P_{ACT}+R_{SLEEP}\cdot P_{SLEEP}}.$$

which are the well-known results for the expected lifetime of an IoT device. Furthermore, the variance of the lifetime of the IoT device becomes:

(22) $$\sigma_{T}^{2}=\frac{B\alpha }{{\left[R_{ACT}\cdot P_{ACT}+R_{SLEEP}\cdot P_{SLEEP}\right]}^{3}}.$$

Diffusion models with and without energy harvesting are quite similar; the difference lies in the choice of diffusion parameters. Furthermore, in the case of energy harvesting, the process may increase and restart after visiting the limiting barriers; therefore, its evolution is more complex. We recently investigated this case and compared diffusion and Markov approach in Reference [8]. To simplify the description, we decided here to limit the model to the case without energy harvesting.

**Figure 5:**
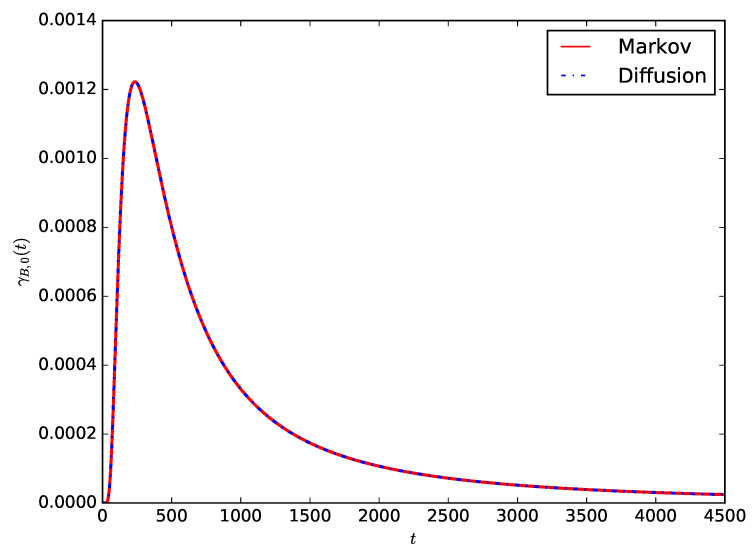
Comparing the probability density of the lifetime of the IoT device $\gamma_{B,0}\left(t\right)$ for B=100 Wh and $P_{D}=0.2$ W, Markov and diffusion models.

Figure 5 shows the comparison of the probability density of the lifetime of the IoT device $\gamma_{B,0}\left(t\right)$ given by Equation (18) in the case of B=100 Wh and $P_{D}=0.2$ W with the corresponding Markov result in Equation (11). For the diffusion model, we use $C_{B}^{2}=1$ to ensure that the distribution of the time required to consume a unit of energy is exponentially distributed to compare the Markovian and diffusion approximation models. It can be observed that the distribution of the lifetime of the IoT device obtained using the proposed diffusion model is the same as that obtained using the pure death Markovian model used in References [6,7] to model the battery of an IoT device.

We may propose a more complex Markovian model by replacing exponential distributions with a squared coefficient of variation equal to one by distributions composed of exponentially distributed phases, e.g., Cox or hyper-Erlang distribution, fit its parameters to approximate any distribution with any desired $C_{B}^{2}$. It is an extensively investigated problem, cf. Reference [58]. The fitting should be achieved numerically, and we dispose of tools allowing it, e.g., Reference [59]. In this case, a state of the Markovian model is represented not only by the quanta of energy in the modelled battery but also by the current phase of service distribution; therefore, the number of states increases. The Chapman–Kolmogorov equations of the corresponding Markov chain can be solved only numerically. That means that the change of $C_{B}^{2}$, which is just a change of parameter value in diffusion model, is possible but tedious in the case of a Markovian model.

The probability that the energy stored in the battery is completely depleted after time *t* is

(23) $$\begin{array}{c} \Gamma_{B,0}\left(t\right)=\int_{0}^{t}\gamma_{B,0}\left(\xi \right)d\xi =\frac{1}{2}\left(e^{-\frac{2B\beta }{\alpha }}\mathrm{erfc}\left[\frac{B-\beta t}{\sqrt{2\alpha t}}\right]+\mathrm{erfc}\left[\frac{B+\beta t}{\sqrt{2\alpha t}}\right]\right). \end{array}$$

where

$$\mathrm{erfc}\left(t\right)=1-\mathrm{erf}\left(t\right),\;\;\;\mathrm{and}\;\;\;\mathrm{erf}\left(t\right)=\frac{2}{\sqrt{\Pi }}\int_{0}^{t}e^{-\xi^{2}}d\xi .$$

During the deployment of the IoT device, it is desired to predict the time after which the battery’s energy level should have decreased to a predefined threshold so that the operator could consider changing the battery or charging it. Suppose that this threshold is x=ηB, where η∈(0,1), then the time *t* after which the energy level of the battery decreased to the defined threshold is the first passage time from x=B at time t=0 to x=ηB at time *t*, and its distribution is

(24) $$\gamma_{B,\eta B}\left(t\right)=\frac{B(1-\eta )}{\sqrt{2\pi \alpha t^{3}}}e^{-\frac{{\left(B\left(1-\eta \right)+\beta t\right)}^{2}}{2\alpha t}}$$

with mean

(25) $$\begin{array}{c} \mu_{t}=\int_{0}^{\infty }t\gamma_{B,\eta B}\left(t\right)dt=-\frac{B(1-\eta )}{\beta }, \end{array}$$

and variance

(26) $$\begin{array}{c} \sigma_{T}^{2}=\int_{0}^{\infty }t^{2}\gamma_{B,\eta B}\left(t\right)dt-\mu_{t}^{2}=-\frac{B(1-\eta )\alpha }{\beta^{3}}. \end{array}$$

The probability that after time *t*, the energy level of the battery should have decreased to reach the defined threshold is

(27) $$\begin{array}{ccc} \Gamma_{B,\eta B}\left(t\right) & = & \int_{0}^{t}\gamma_{B,\eta B}\left(\xi \right)d\xi =\\ & = & \frac{1}{2}\left(e^{-\frac{2B(1-\eta )\beta }{\alpha }}\mathrm{erfc}\left[\frac{B(1-\eta )-\beta t}{\sqrt{2\alpha t}}\right]+\mathrm{erfc}\left[\frac{B(1-\eta )+\beta t}{\sqrt{2\alpha t}}\right]\right). \end{array}$$

## 5. Analysis of Ghost Energy Depletion Attacks on an IoT Network

A ghost energy depletion attack (GEDA) is one in which an adversary masquerades as a trusted device and compels other IoT devices within the network to perform unnecessary computational and communication operations to quickly deplete the energy stored in the battery of the victim devices and eventually shut down the devices. Two primary forms of ghost energy depletion attacks are the high computational load on device GEDA and the MAC misbehaviour GEDA. In the high-computational-load GEDA, the adversary overwhelms its victims with bogus messages to quickly drain the energy stored in their batteries. Even though it is easier to detect these attacks with attack detectors, a ghost attacker may cleverly conduct such an attack by sending messages at different times or addresses to a subset of victim devices in its range [46]. In a MAC misbehaviour GEDA, a ghost attacker deliberately abuses the MAC protocol (e.g., CSMA/CA protocol) to create collisions on the shared wireless channel to cause other devices within its interference range to consume more energy (quickly draining their batteries) and to deprive them of accessing the channel. In analysing GEDAs, it is essential to take note of the factors that influence such attacks, which includes:

1 The energy consumption of the various hardware components of the IoT device (e.g., microcontrollers, radio transceivers, sensors, actuators, and other electronic components). Energy-demanding microcontrollers and radio transceivers will consume more energy than energy-efficient ones. They will drain the energy stored in the battery of the IoT device quickly during a battery depletion attack.
2 The energy capacity of the IoT battery. For a given IoT device, the lifetime depends mainly on its battery’s energy capacity. With a high-capacity battery, the lifetime of the device could be longer. A ghost energy depletion attack will quickly shut down a device with a small battery capacity.
3 Frequency of data collection (sensing), actuation (where necessary), processing, and communication (reception and transmission of information). The more frequent the device’s sensing, processing, actuation, processing, and communication operations, the higher the device’s energy consumption. A ghost attacker could compel victim IoT devices to perform such operations more frequently than during normal operations.
4 The MAC protocol in the link layer. A ghost attacker can abuse the MAC protocol in the link layer to create collisions, thus increasing the energy consumption of the devices sharing the channel with it. A collision-free protocol at the link layer could reduce this kind of attack.
5 The cryptographic algorithm is implemented on the IoT device to encrypt and decrypt information. The energy required to encrypt or decrypt a packet depends on the number of microcontroller clock cycles required to execute the algorithm (encryption or decryption) and the average current drawn by each cycle. Therefore, with information about the number of cycles required to execute the algorithm, the current drawn in each cycle, the microcontroller’s clock frequency, and the microcontroller’s operating voltage, the energy required to execute an encryption or decryption algorithm on an IoT device can be estimated. The more sophisticated or computationally intensive the cryptographic algorithm, the more quickly it can be leveraged by an attacker to drain the energy of an IoT device.
6 The packet sizes. The longer the packet size, the more energy is required to transmit the packet and the longer the time required to transmit the packet. A ghost attacker could decide to create longer packets that take too long to transmit, causing other IoT devices sharing the channel with it to experience more collisions.

### 5.1. Analysis of High Computational Load Ghost Energy Depletion Attack

Consider an IoT device in an IoT network with *N* nodes. Suppose that the device is working in a duty-cycling mode with a duty cycle of D=τ/T, where τ is the duration of the active period and *T* is the length of the cycle. Within the active period, the device can receive and decrypt a packet or encrypt and transmit a packet. If the device completes the reception or transmission of packets and the active period is not yet finished, the radio and the microcontrollers could be switched to a low-power mode. If no packet arrives or there is no packet to transmit, the radio is turned off, and the microcontrollers are switched to a deep sleep mode (where it consumes a tiny amount of power). After a period $T_{s}$, the device wakes up again to either receive or transmit packets.

Suppose a ghost attacker crafts bogus packets and sends them to the victim device to force it to spend energy to receive and perform security checks (e.g., access control, message integrity checks, and decryption). The access control mechanism is based on the principle that after receiving a packet, the device compares its source address with a list of valid addresses. If there is a match, the packet is accessed; otherwise, it is rejected. If the ghost attacker can masquerade as a legitimate device, its packets might be accepted by the victim device. However, after performing a message integrity check or decrypting the message, it will realise that it will fail. Although the security checks eventually failed and the packet from the ghost attacker dropped, the device must have spent a significant amount of energy performing some computation.

Suppose that a ghost attacker sends bogus packets to the victim device at a mean rate of κ and the mean arrival rate of packets to the victim device from both attack and legitimate sources is γ=κ+ν, where ν is the mean arrival rate of normal packets from legitimate sources. If, within a given active period, the device receives $N_{r}$ packets (both attack and normal ones) and performs security checks for these packets, then the energy consumed by the microcontroller during the process of receiving the packet and executing the security algorithms to perform the security checks is [46]:

(28) $$E_{comp}^{rx}=N_{r}\left(T_{dec}P_{MCU}^{a}+T_{rx}P_{MCU}^{i}\right),$$

where $T_{dec}$ is the time required to perform the security checks (access control, decryption, and integrity verification) for a given packet, which depends on the security mechanism that is implemented, $T_{rx}$ is the time required to receive a packet (which depends on the size or length of the packet), and $P_{MCU}^{a}$ is the power drawn by a microcontroller unit (MCU) when it is in the active mode and the power drawn by the MCU when it is in the idle mode. The energy required to execute the algorithms required to perform the security checks (including decryption and MIC verification) after receiving the packet can be given by

(29) $$E_{sec}=\frac{N_{c}I_{sec}V_{sec}}{f},$$

where $N_{c}$ is the number of clock cycles required to perform the security checks, $I_{sec}$ is the average current drawn by each clock cycle, $V_{sec}$ is the operating voltage of the MCU, and *f* is the clock frequency of the MCU. Therefore, the more attack packets that are successfully received by the victim device, the higher the amount of energy wasted executing the security check algorithms (wasting MCU clock cycles to perform security computations).

The energy consumed by the radio module in receiving both the attack and normal packets within a given active period is

(30) $$E_{rx}=\left\{\begin{array}{ccc} N_{r}\left(T_{dec}+T_{rx}\right)P_{rx} & \;\;\;\;\;\;\;\;N_{r}\left(T_{dec}+T_{rx}\right)\geq \tau \;, & \\ \tau P_{rx} & \;\;\;\;\;\;\;\;\mathrm{otherwise}, \end{array}\right.$$

where $P_{rx}$ is the power required to receive a single packet.

Suppose an attacker compromises an IoT device and then reconfigures it to perform more sensing (measurement) operations more frequently than required. The packets that belong to the extra measurement can be considered attack packets because the device spends energy to sense, encrypt, and transmit the packets. Suppose that the victim device performs $N_{t}$ number of transmission (including the transmission of packets from necessary and unnecessary measurements), then the energy consumed by the MCU in performing cryptographic operations (including encryption of the packets) and transmitting the packets is given by

(31) $$E_{comp}^{tx}=N_{t}\left(T_{enc}P_{MCU}^{a}+T_{tx}P_{MCU}^{i}\right).$$

where $T_{enc}$ is the time required to encrypt a packet and $T_{tx}$ is the time required to transmit a packet. The energy required to perform the cryptographic operations (including encryption) before transmitting the packet is similar to Equation (29). The amounts of energy consumed by the radio module in the transmission of both attack and normal packets within an active period, assuming that there are no collisions, are

(32) $$E_{tx}=\left\{\begin{array}{ccc} N_{t}\left(\eta P_{t}+P_{o}\right)\left(T_{enc}+T_{tx}\right) & \;\;\;\;\;\;\;\;N_{t}\left(T_{enc}+T_{tx}\right)\geq \tau \;, & \\ (\eta P_{t}+P_{o})\tau & \;\;\;\;\;\;\;\;\mathrm{otherwise}, \end{array}\right.$$

where η is the conversion factor of the power amplifier from electric power to RF power and $P_{o}$ is the electronic power consumption overhead.

### 5.2. Analysis of MAC Misbehaviour Ghost Energy Depletion Attack

Compromised IoT devices could be exploited to create and generate attack packets and to cause collisions in the shared channel. An increase in collisions in the channel will lead to an increase in the energy consumption of the IoT devices in the network. The mean total effective traffic intensity in the channel is

(33) $$\lambda =N\lambda_{0}\frac{1}{1-P_{c}}+\lambda_{A},$$

where $P_{c}$ is the probability that a device experiences a collision when it tries to transmit a packet and $\lambda_{0}$ is the mean arrival rate of packets at the output transmission queue of an IoT device. The first right term in Equation (33) is the effective normal traffic intensity created in the channel by IoT devices that are behaving normally, and the last term $\lambda_{A}$ is the additional mean traffic intensity created by compromised IoT devices (we also refer to it as the attack traffic intensity).

**Figure 6:**
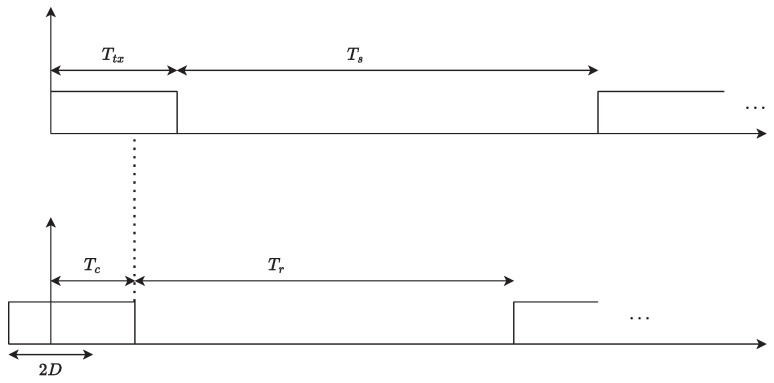
Channel access behavioral model of the IoT device.

All IoT devices are listening to the channel in order to sense when the channel is free so that they can transmit their packets. Whenever the channel is free, an IoT device can transmit its packet normally within the timeframe of $T_{tx}$, as shown in Figure 6. Suppose that a device transmits all its packets, and its buffer is empty. In that case, it switches to a low-power mode until the next trial (when it has a packet to transmit or there is an incoming packet). It will wake up to either receive messages from the IoT gateway or take measurements and transmit them to the gateway. The more time a device spends in the low-power mode, the longer its lifetime. However, if a device detects a collision, it will try to access the channel again after a backoff time or retransmission time $T_{r}$, proportional to 1/λ. The probability that there is a collision in the channel is

(34) $$P_{c}=1-e^{-2\lambda D},$$

where *D* is the propagation delay of the wireless communication channel between the IoT device and the IoT gateway. By substituting $P_{c}$ in Equation (33) and simplifying, we have

(35) $$e^{2\lambda D}=\frac{\lambda -\lambda_{A}}{N\lambda_{0}}=\frac{\lambda }{N\lambda_{0}}-\frac{\lambda_{A}}{N\lambda_{0}},$$

shown in Figure 7.

The mean number of transmission attempts, including successful transmissions, is

(36) $$N_{t}=\sum_{n=1}^{\infty }nP_{c}^{n-1}\left(1-P_{c}\right)=\frac{1}{1-P_{c}},$$

and the mean number of collisions in the channel is

(37) $$N_{c}=\frac{1}{1-P_{c}}-1=\frac{P_{c}}{1-P_{c}}.$$

Therefore, the service time at the output queue of the IoT device is

(38) $$\begin{array}{c} \frac{1}{\mu }=\frac{P_{c}}{1-P_{c}}\left(T_{c}+T_{r}\right)+T_{tx}=\left[e^{2\lambda D}-1\right]\left(T_{c}+T_{r}\right)+T_{tx}. \end{array}$$

The mean time required to transmit an IoT packet of size *m* is

(39) $$T_{tx}=\frac{m}{C},$$

where *C* is the Shanon capacity of the IoT wireless channel given by

(40) $$C=W{log}_{2}\left(1+SNR\right),$$

where *W* is the channel bandwidth, $SNR=P_{r}/\left(P_{I}+P_{N}\right)$ is the signal-to-noise ratio, and $P_{r}$, $P_{I}$, $P_{N}$ are the received power, the interference power, and the noise power, respectively. The channel attenuates the power level of the signal transmitted by the IoT device, and the power that is received by the IoT wireless access point is

(41) $$P_{r}=P_{L}\cdot P_{t}.$$

$P_{t}$ is the transmit power, and the path loss $P_{L}$ is given by [1]

(42) $$P_{L}=L_{0}hd^{-a},$$

where *d* is the distance between the IoT device and the wireless access or gateway, *h* is a random variable that represents the fading in the channel, *a* is the path-loss exponent, and $L_{0}$ is a constant determined by the antenna gain, radio frequency, and the propagation environment. More advanced radio network planning models could be used to determine the path loss to account for possible environmental obstacles which cause radio signal degradation or fading. The retransmission delay is assumed to be exponentially distributed with parameter λ, and the mean retransmission delay $T_{r}$ is proportional to 1/λ. Furthermore, we assume that the collision time is uniformly distributed between 0 and *D*, and the mean collision time $T_{c}$ is, therefore, D/2. Thus, Equation (38) becomes

(43) $$\frac{1}{\mu }=\left[e^{2\lambda D}-1\right]\left(\frac{1}{D}+\frac{1}{\lambda }\right)+\frac{m}{W{log}_{2}\left(1+\frac{L_{0}hd^{-a}P_{t}}{P_{I}+P_{N}}\right)}.$$

For most IoT devices, the majority of the communication is in the uplink (from the IoT device to the access point), especially for IoT devices whose function is to take measurements (sensing) and send them to the fog or cloud servers. Since most of the communication is in the uplink, an attacker can easily conduct energy depletion attacks by causing the IoT device to increase its transmit power, increasing the time required to transmit a packet (by increasing packet sizes *m* or creating interference in the wireless channel) or by creating collisions in the shared wireless channel.

It should be noted that by deciding to generate or create packets with larger sizes, the ghost attacker increases the transmission times, thereby increasing the likelihood of collisions in the channels. Furthermore, by increasing its traffic (by increasing its measurement frequency), the ghost attacker triggers an increase in λ, which increases the likelihood of collisions in the channel and compels other devices sharing the channel to consume more energy, hence, quickly draining their batteries.

**Figure 8:**
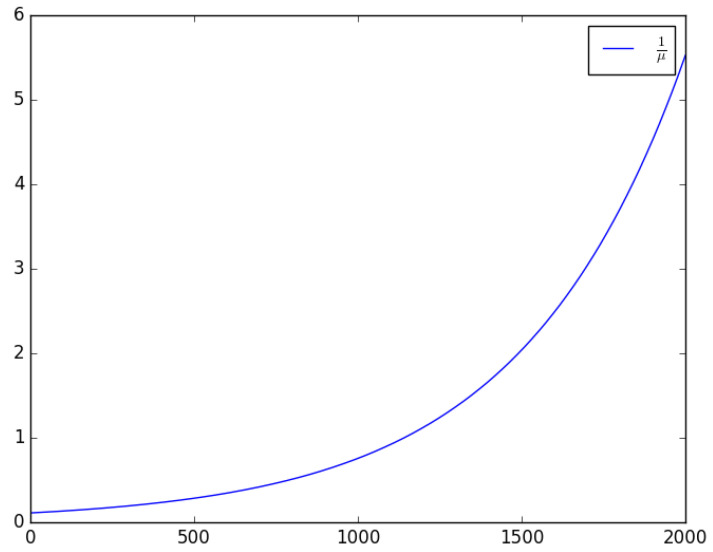
The relationship between the mean active time of the transmitter and the mean total effective traffic intensity in the channel: 1/μ vs. λ, for D=0.001, $T_{c}=0.001$, $T_{r}=0.1$, $T_{tx}=0.01$, and λ∈[0,2000].

Increasing the number of collisions in the shared wireless channel will increase the mean active time of the transmitter. The relationship between the mean active time of the transmitter and the mean total effective traffic intensity in the channel, λ is shown in Figure 8. The mean total effective traffic intensity in the channel, λ, can be increased by increasing the attack traffic. The attacker’s main objective is to maximise the active period, that is, to increase the time that the microcontroller unit and the radio unit spend performing computation and reception or transmission operations, respectively. By maximising the active period of the MCU and radio unit, more energy is drained from the battery and quickly depletes its energy content.

## 6. Numerical Examples

We present some numerical examples to study the dynamics of the energy depletion process in a small-sized battery used to power IoT devices. We present the influence of design parameters that can be selected during the design and deployment of the IoT devices on the time it takes to completely drain all of the energy stored in the battery. This time gives the lifetime of the IoT device from the time the device is deployed to when the energy stored in its battery is completely drained and the device is shut down.

**Figure 9:**
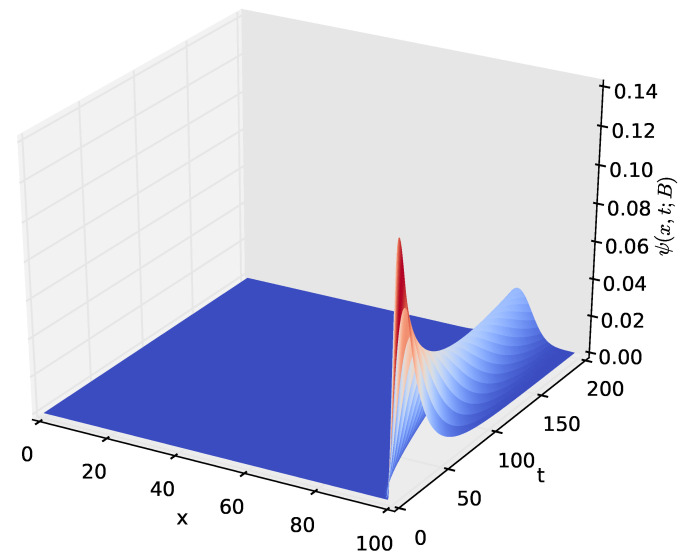
The distribution ψ(x,t;B), of the amount of energy *x* present in the battery at time *t*, for t∈(0,200] and x∈[0,100].

**Figure 10:**
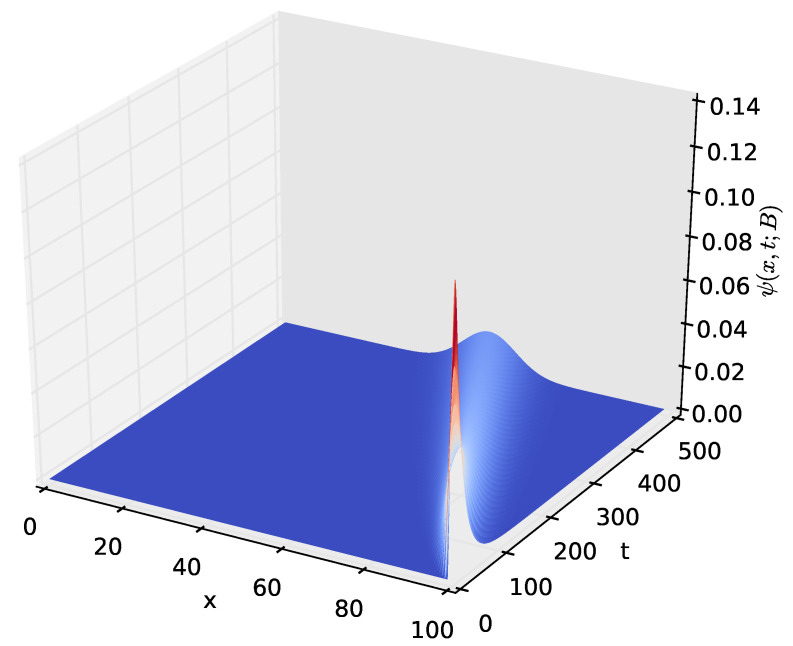
The distribution ψ(x,t;B), of the amount of energy *x* present in the battery at time *t*, for t∈(0,500] and x∈[0,100].

**Figure 11:**
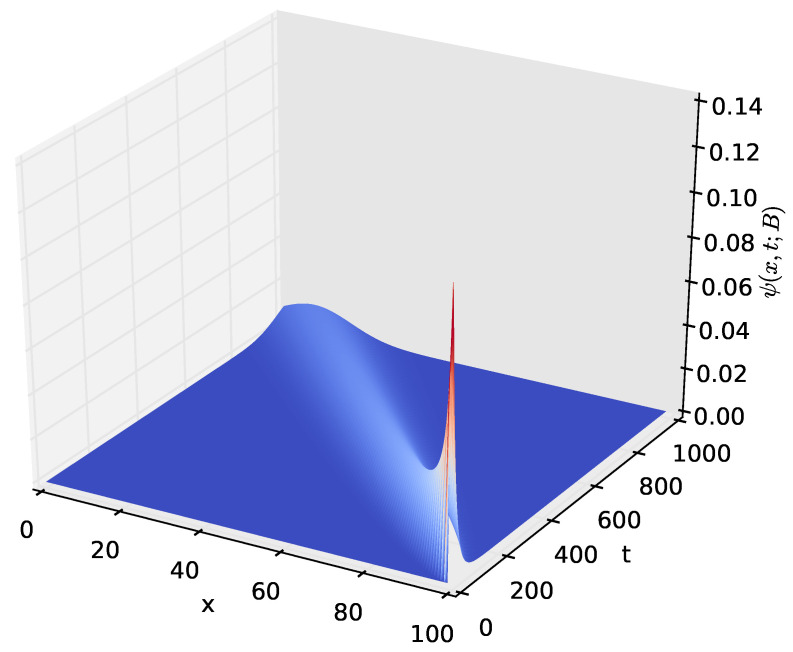
The distribution ψ(x,t;B), of the amount of energy *x* present in the battery at time *t* for t∈(0,1000] and x∈[0,100].

**Figure 12:**
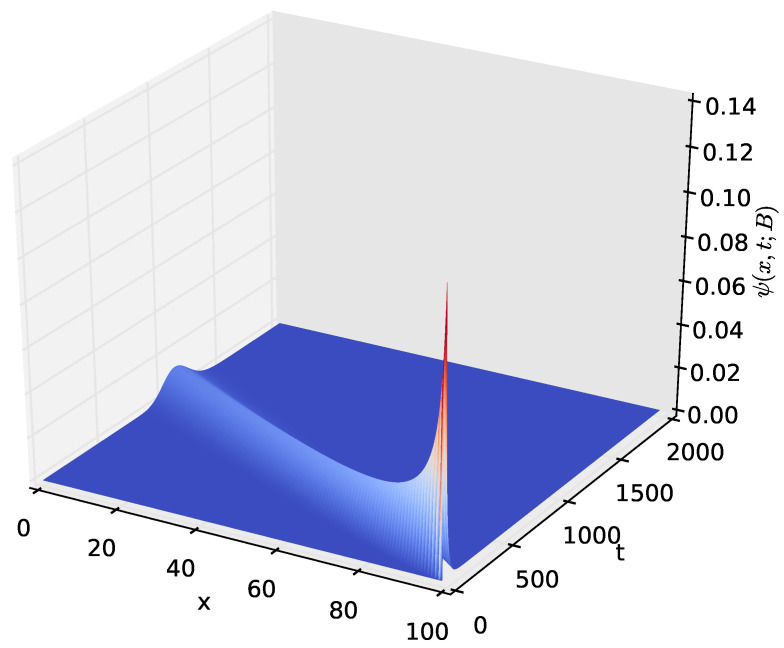
The distribution ψ(x,t;B), of the amount of energy *x* present in the battery at time *t* for t∈(0,2000] and x∈[0,100].

Figure 9 shows the probability density of having the amount *x* of energy in the battery at time *t* given that the discharging process started with x=B amounts of energy in the battery. After a certain time *t*, the distribution of the energy present in the battery decreases to x=0, which happens when the energy stored in the battery is completely depleted. The time axis gives the distribution of time required for the amount of energy present in the battery to decrease from x=B to a given amount *x*. In order to observe how the distribution of the amount of energy in the battery varies over time, we increased the time axis, as shown in Figure 10, Figure 11 and Figure 12. For the results presented in Figure 9, Figure 10, Figure 11 and Figure 12, $P_{A}=0.12471$ W, $P_{S}=0.090$ W, and $R_{SLEEP}=0.95$, and $R_{ACT}=0.05$, $CB^{2}=1$, and B=100.

**Figure 13:**
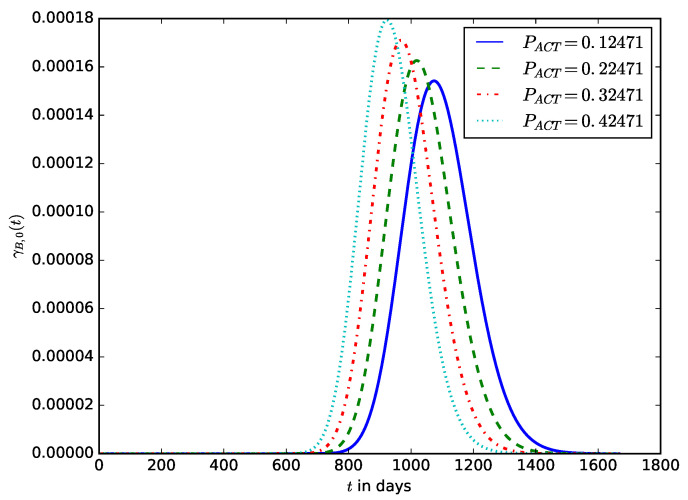
The influence of the active mode power, $P_{ACT}$, on the distribution of the lifetime of the IoT device.

Figure 13 shows the influence of the average power consumed by the device in the active mode, $P_{ACT}$, on the distribution of the device’s lifetime. It can be observed that a minimal increase in the average power consumed by an IoT device in active mode can significantly reduce the lifetime of the IoT device. In battery depletion attacks, an attacker could cause the IoT device to increase its transmission power, increasing the power consumed by the IoT device in active mode. There are various ways in which the power consumed by the device when it is in active mode can be increased. For the plot in Figure 13, B=100, $P_{S}=0.090$ W, and $R_{SLEEP}=0.95$, and $R_{ACT}=0.05$, $CB^{2}=1$.

**Figure 14:**
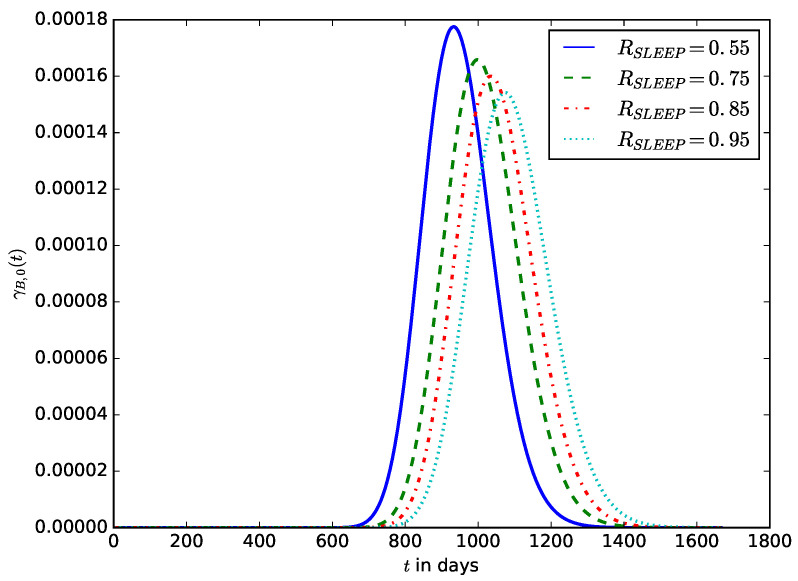
The influence of the proportion of sleep time, $R_{SLEEP}$, on the distribution of the lifetime of the IoT device.

Figure 14 shows the influence of the proportion of sleep time, $R_{SLEEP}$, on the distribution of the lifetime of an IoT device. The distribution is shifted to the right as $R_{SLEEP}$ increases. This is because increasing sleep time reduces the energy consumed by the IoT device and increases its lifetime. The most popular energy depletion attacks designed to completely drain the energy of the battery of the IoT device are denial of sleep and various types of vampire attacks. They are conducted by manipulating some device or network parameters to reduce the device’s sleep time (and hence, $R_{SLEEP}$).

**Figure 15:**
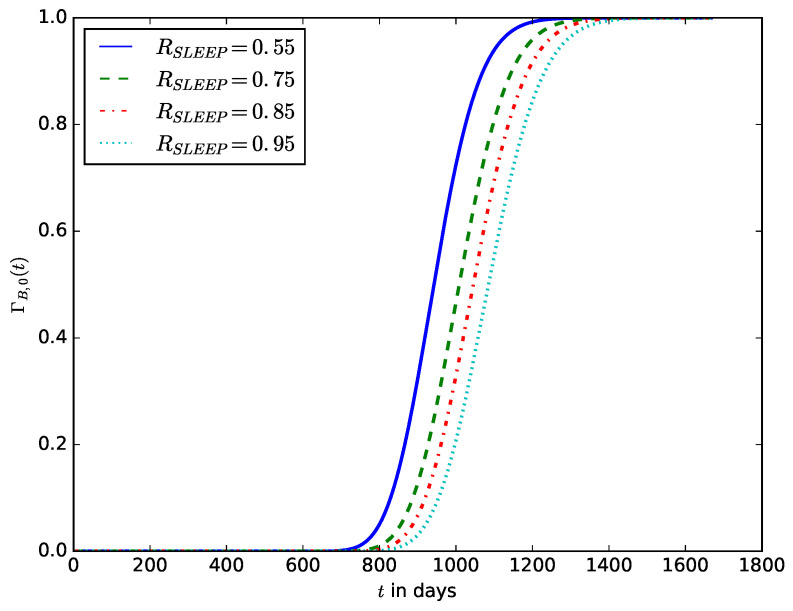
The influence of the proportion of sleep time, $R_{SLEEP}$, on the probability that the energy stored in the battery is completely depleted.

Figure 15 shows the influence of the proportion of sleep time, $R_{SLEEP}$, on the probability that the energy stored in the battery is completely depleted before a given time *t*. It shows that as the proportion of sleep time increases, the higher the probability that the energy stored in the battery will be completely depleted before a defined time *t*.

**Figure 16:**
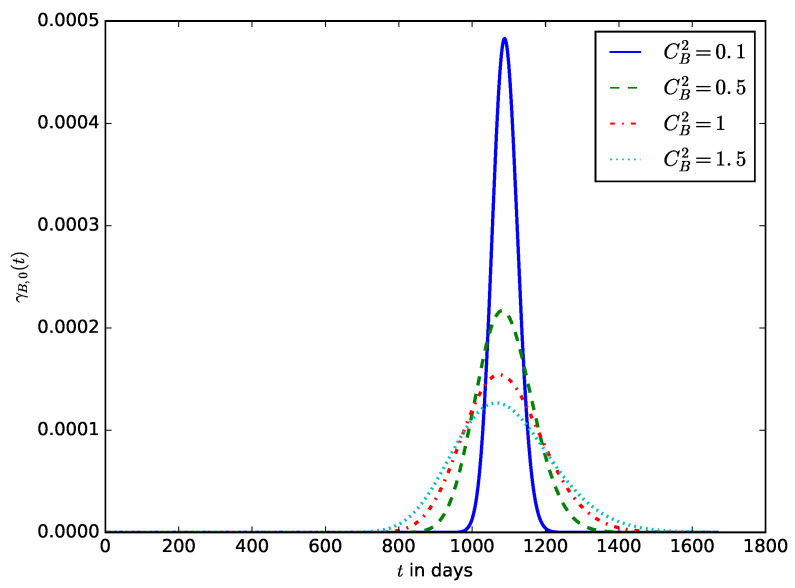
The influence of the squared coefficient of variance of the energy consumption $C_{B}^{2}$ on the distribution of the lifetime of the IoT device.

Figure 16 influence of the coefficient of variance of the energy consumption $C_{B}^{2}$ on the distribution of the lifetime of the IoT device. It shows that significant variations in the energy consumption of the IoT also result in a considerable variation in the lifetime of the IoT devices and makes it difficult to predict the lifetime of the IoT device or the expected time to change the device’s battery. Usually, the energy consumption of IoT devices may vary very slightly, but the variations could be significant if the device experiences energy depletion attacks at random times. Some energy depletion attacks that do not degrade the QoS (some improve QoS by increasing the transmit power when the noise or interference level is high while draining more energy from the battery) are therefore difficult to detect using the traditional QoS-based attack detection mechanisms. In this case, it could be preferable to monitor both the QoS and the energy consumption metrics and use them for attack detection.

**Figure 17:**
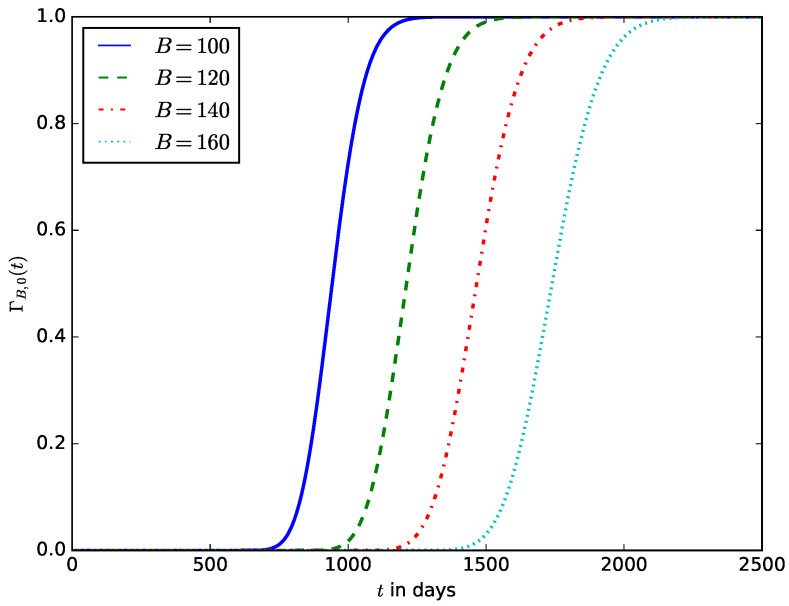
The influence of the battery capacity *B* on the probability that the energy stored in the battery is completely depleted.

**Figure 18:**
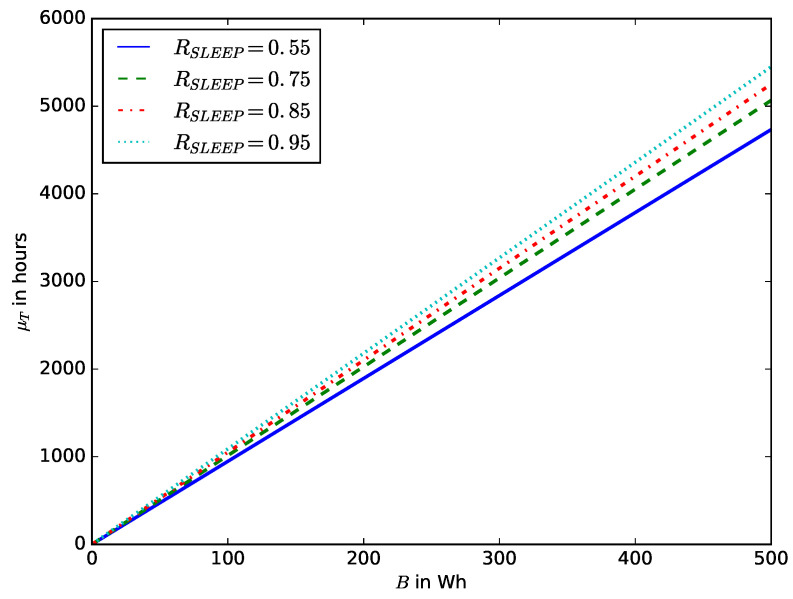
Mean device lifetime $\mu_{T}$ versus battery capacity *B* for various values of sleep mode ratio $R_{SLEEP}$.

Figure 17 shows the influence of the battery capacity on the probability that before time *t* the energy stored in the battery is completely depleted. The IoT devices that require a long life include those used in industries such as oil and gas, agriculture, health care, wildlife conservation, forestry, and water monitoring [60]. Using batteries with small energy storage capacity will result in frequent battery replacements, increasing maintenance costs. However, an IoT device’s choice of battery capacity depends on the battery’s cost, size, weight, and energy density. Therefore, based on the power consumption budget of the IoT device, the battery specifications should be selected in such a way as to have a long lifetime. Figure 18 shows the relationship between the battery capacity and the mean lifetime of the device.

**Figure 19:**
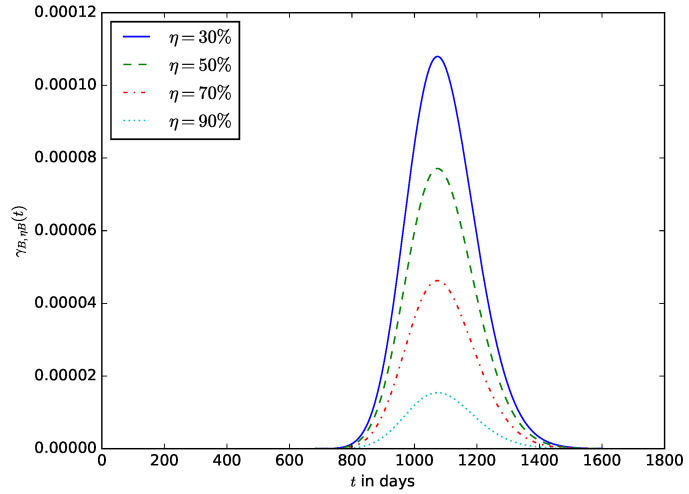
The probability density function $\gamma_{B,\eta B}\left(t\right)$ that after time *t*, the amount of energy present in the battery is x=ηB; that is, the battery must have discharged to 1−η percent of its initial amount of energy.

**Figure 20:**
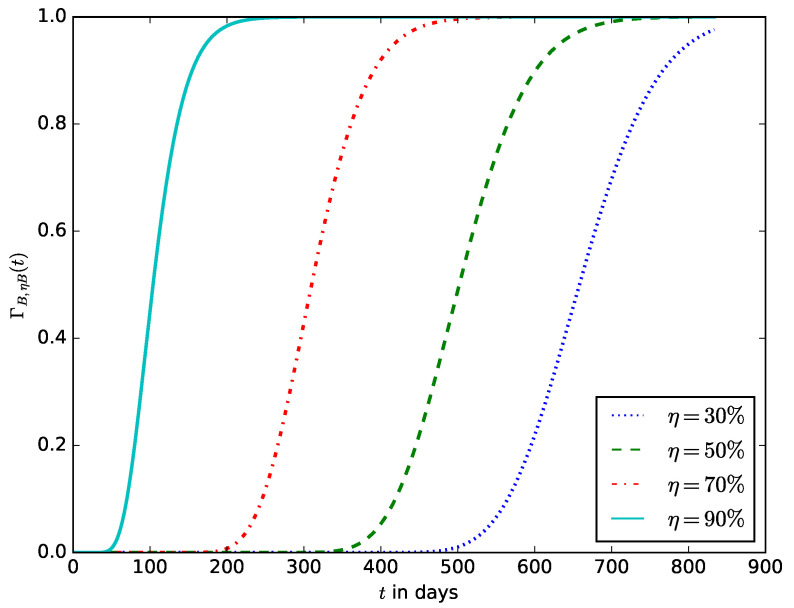
The probability $\Gamma_{B,\eta B}\left(t\right)$ that after time *t*, the amount of energy present in the battery is x=ηB; that is, the battery must have discharged to 1−η percent of its initial amount of energy.

When deploying an IoT device in an IoT network, the probability of the time after the energy stored in the battery should have decreased to a defined percentage (η%) of its initial amount. This enables the operator to estimate the time after the IoT device’s battery should be replaced without waiting until the energy stored in the battery is completely depleted before the battery is replaced. It also ensures that the device is not shut down due to the complete depletion of the energy stored in the battery. Figure 19 shows the influence of the energy threshold percentage x=η on the probability density function, $\gamma_{B,\eta B}\left(t\right)$. It is the pdf that after time *t*, the energy in the battery is x=ηB; that is, the battery must have discharged to 1−η percent of its initial amount of energy. Figure 20 shows the influence of the energy threshold percentage x=η, probability $\Gamma_{B,\eta B}\left(t\right)$ that after time *t*, the amount of energy present in the battery is x=ηB; that is, the battery must have discharged to 1−η percent of its initial amount of energy.

## 7. Conclusions

During the development and deployment of IoT devices and networks, making reasonable tradeoffs between QoS, security, and energy consumption is essential to ensure reliability, security, and a longer lifetime of IoT devices. Prioritising power consumption when designing and deploying IoT devices and networks is essential. Some of the ways to reduce power consumption include the implementation of sleep mode (which could reduce up to 90% of the energy consumption), avoiding excessive communication requests, choosing when and how to transmit information, selecting the most appropriate wireless protocol [60], and implementation of energy depletion attack systems to prevent attacks that are aimed at rapidly draining the battery of the device. Energy harvesting has been used to recharge the battery with energy harvested from the environment to prolong the lifetime of IoT devices and minimise the impact of battery depletion. Our study was limited to developing the diffusion approximation for a battery without any renewable energy source.

We have applied a diffusion or Brownian motion process to model the energy depletion process of a battery of an IoT device. We used the model to obtain the probability density function, mean, variance, and probability of the lifetime of an IoT device. Furthermore, we studied the influence of active power consumption, sleep time, and battery capacity on the probability density function, mean, and probability of the lifetime of an IoT device. Since battery depletion attacks are always aimed at manipulating the IoT device to increase its energy consumption significantly, the numerical examples enabled us to study the influence of battery depletion attacks on the distribution of the lifetime of an IoT device. We also introduced in our model the energy threshold after which the device’s battery should be replaced to ensure that the battery is not completely drained before it is replaced. The time after which the battery should be replaced can be obtained from our model. Therefore, the diffusion approximation can be used to conveniently model the energy depletion process of the battery of an IoT device. Thus, with knowledge of the battery capacity, the average power consumption, and the variance of energy consumption (if any), the probability density function, the mean, variance, and probability of the lifetime of an IoT device can be obtained.

## Figures and Tables

**Figure 7:**
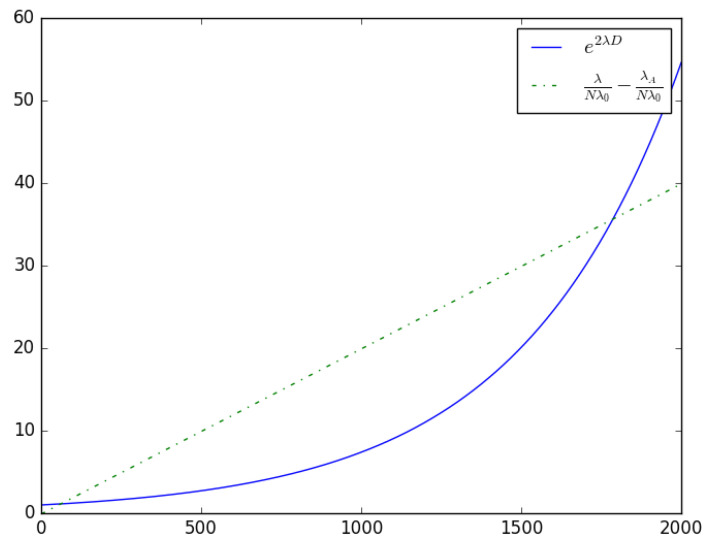
An illustration of Equation (35) with $\lambda_{A}=5$, $\lambda_{0}=1$, D=0.001, N=50, λ∈[0,2000]; the intersection points indicate the operating points of the system.

## Data Availability

Not applicable.
